# Impact of the first COVID-19 shutdown on patient volumes and surgical procedures of a Level I trauma center

**DOI:** 10.1007/s00068-021-01654-8

**Published:** 2021-04-21

**Authors:** Carolin A. Kreis, Birte Ortmann, Moritz Freistuehler, René Hartensuer, Hugo Van Aken, Michael J. Raschke, Benedikt Schliemann

**Affiliations:** 1 Department of Trauma, Hand and Reconstructive Surgery, University Hospital Muenster, Albert-Schweitzer-Campus 1, Building W1, 48149 Muenster, Germany; 2 Department of Medical Management and Medical Controlling, University Hospital Muenster, Muenster, Germany; 3 University Hospital Muenster, Muenster, Germany

**Keywords:** SARS-CoV-2, COVID-19, Shutdown, Pandemic, Level I trauma center, Emergency operation

## Abstract

**Purpose:**

In Dec 2019, COVID-19 was first recognized and led to a worldwide pandemic. The German government implemented a shutdown in Mar 2020, affecting outpatient and hospital care. The aim of the present article was to evaluate the impact of the COVID-19 shutdown on patient volumes and surgical procedures of a Level I trauma center in Germany.

**Methods:**

All emergency patients were recorded retrospectively during the shutdown and compared to a calendar-matched control period (CTRL). Total emergency patient contacts including trauma mechanisms, injury patterns and operation numbers were recorded including absolute numbers, incidence proportions and risk ratios.

**Results:**

During the shutdown period, we observed a decrease of emergency patient cases (417) compared to CTRL (575), a decrease of elective cases (42 vs. 13) and of the total number of operations (397 vs. 325). Incidence proportions of emergency operations increased from 8.2 to 12.2% (shutdown) and elective surgical cases decreased (11.1 vs. 4.3%). As we observed a decrease for most trauma mechanisms and injury patterns, we found an increasing incidence proportion for severe open fractures. Household-related injuries were reported with an increasing incidence proportion from 26.8 to 47.5% (shutdown). We found an increasing tendency of trauma and injuries related to psychological disorders.

**Conclusion:**

This analysis shows a decrease of total patient numbers in an emergency department of a Level I trauma center and a decrease of the total number of operations during the shutdown period. Concurrently, we observed an increase of severe open fractures and emergency operations. Furthermore, trauma mechanism changed with less traffic, work and sports-related accidents.

## Introduction

In Dec 2019, the Chinese government first reported a new viral disease called COVID-19 caused by a new viral type SARS-CoV-2 followed by a worldwide pandemic with exponential increase of infection rate and serious impact on our healthcare systems, economy, society and social life [1]. To monitor the development of viral spread and infection, the reproduction value (r value) was recorded by the Robert Koch Institute (RKI), Germany. This value indicates how many people are infected by one infected person and was announced to be *r* = 2.34 on Mar 3rd [2].

Due to the pandemic process, the German Government declared a temporary shutdown as necessary to control the infections process, which started on Mar 16th 2020. The major reason and objective for these serious restrictions was on the one hand the control of the infection process and on the other hand, the warranty of sufficient medical care including sufficient intensive care capacities. The temporary shutdown affected all major social institutions: schools, children`s day care institutions, universities, sport clubs and all public leisure institutions, restaurants, bars and nightclubs, theaters and non-essential institutions. Major events were cancelled, and social contacts were restricted to a maximum of two households meeting at one time. The healthcare system was obliged to only treat emergencies if possible [3]. On Apr 15th 2020, the German government allowed easing of restrictions with corresponding precautionary measures in compliance with hygiene rules [4].

A nationwide shutdown with all its consequences probably leads to changes in daily life of the population and in economy. Against this background, the question arises whether restrictions, quarantine and isolation led to fundamental changes in daily life and whether there is an impact on trauma numbers, mechanisms and injury patters as well as on operation numbers in a Level I trauma center.

A previous analysis by Haffner et al. showed considerable economical and personnel loss in orthopedic and trauma departments of university hospitals in Germany: the COVID-19 pandemic led to a reduction of operation capacity of 49.4%, a loss of expected income of 29.3% and a redistribution of specialist staff of 14.7% [5]. Similar results were found by von Dercks et al. [6]. Their findings demonstrated a reduction of patient cases, a decrease of hospitalized patients and a reduction of occupancy days during the first 7 weeks of the COVID-19 pandemic. Some findings point out the influence on population and a rise of domestic violence and emotional stress, depression, fears, unemployment and reduced income [7, 8]. Data concerning trauma, injury patterns and operation numbers are limited [9, 10] but required to understand the consequences of a shutdown in the face of a pandemic.

The aim of our study was to analyze trauma numbers and mechanisms as well as surgical procedures in case of a shutdown due to the occurrence of a pandemic. This study might help to draw consequences for further states of emergencies to coordinate health care systems as the “second pandemic wave” has just “arrived” and an end of the pandemic is not yet in sight. Therefore, we analyzed retrospectively the total and daily numbers of emergency patient contacts including trauma mechanisms and injury patterns as well as operation numbers in a Level I trauma center in Germany during the 35-day period of the COVID-19 shutdown. Findings were compared to a calendar-matched control period (CTRL) in 2019, to a 15-day pre-shutdown transition and a 15-day post-shutdown transition before and after shutdown in Mar/Apr 2020.

## Methods

We analyzed retrospectively all emergency patient records and all operation records of the trauma emergency department of a German Level I trauma center. We evaluated and compared the following time periods: Mar 16th 2019 until Apr 19th 2019 and Mar 16th 2020 until Apr 19th 2020.

Finally, we defined four investigation time periods according to the German Infection Protection Act:

1. Calendar-matched control time (CTRL): Mar 16th 2019–Apr 19th 2019 (35 days)
2. Pre-shutdown time (PREST): Mar 1st 2020–Mar 15th 2020 (15 days)
3. Shutdown time (SHUTDOWN): Mar 16th 2020–Apr 19th 2020 (35 days)
4. Post-shutdown time (POST): Apr 20th 2020–May 4th 2020 (15 days)

All data were taken from the hospital’s clinical information system (ORBIS, Dedalus, Health Care, Bonn, Germany). Ethical approval was obtained from the clinical ethics committee (reference number: AZ 2020-397-f-S). Documentation was filtered by epidemiological, clinical and therapeutic parameters.

### Data analysis

Collected data were transferred to IBM SPSS Statistics program for statistical analysis (IBM Corp. Released 2020. IBM SPSS Statistics for Windows, Version 27.0. Armonk, NY: IBM Corp). For statistical analysis, we used descriptive statistical methods with a 95% confidence interval (CI), which was not adjusted for multiple testing. Therefore, statistical analysis is to be evaluated as explorative with no statistical significance. To capture the total number of ambulant patient cases and operation cases, the cumulative number of cases per day was registered in each of the four investigation time periods. Daily fluctuations were plotted as a calendar time function. A visual shift is to be justified by the year 2020 being a leap year.

Daily cumulative patient cases of the trauma emergency department and numbers of operations of the aforementioned 4 time periods were compared with using incidence rate ratios (IRR). Operations are conducted standardly concerning the Amsterdam Operation Criteria [11]. Daily cumulative cases were determined as an addition of daily treatment divided by the number of days of the corresponding time period. A negative binomial regression analysis defined the IRR and the corresponding 95% CI. In addition, we analyzed further variables concerning trauma mechanisms and treatments between the CTRL and the shutdown period. The incidence of a variable was defined as numbers of each variable divided by the number of days within respective periods. To compare the CTRL and shutdown period, we calculated risk ratios (RR), quotient from incidence values of the shutdown period and the CTRL and 95% CI via crosstab and pairwise comparison. Data were plotted as Forest plots by Microsoft Excel (Microsoft Corporation. Microsoft Excel for Microsoft 365 MSO, Version 2009. Redmond, Washington: Microsoft Corp).

Death numbers were registered as a cumulation in between 24 h and during hospitalization. To compare the degree of severity of trauma, the ISS-Score was recorded. Demographic data concerning patients` professions were documented numerically.

According to the German DRG-System, Case mix indexes were also recorded for the CTRL and shutdown period to indicate the average case severity.

## Results

**Fig. 1:**
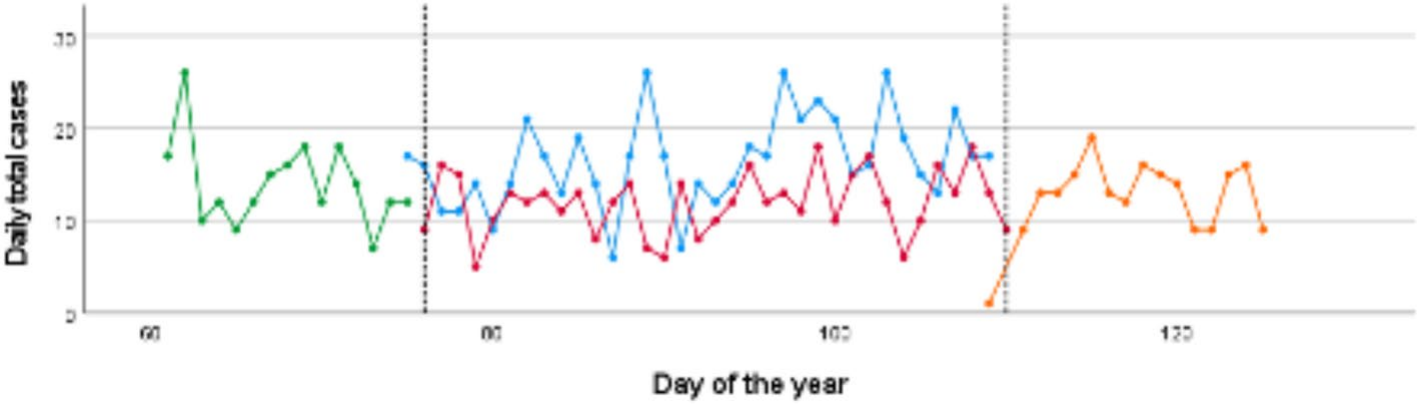
Development of daily total trauma cases before, during and after the COVID-19 shutdown. The solid lines describe the development in the CTRL-period (blue) in 2019, pre-shutdown (green), shutdown (red) and post-shutdown (orange) period in 2020. Dotted lines indicate the beginning and end of the shutdown period (first line: Mar 16th 2020; second line: Apr 20th 2020)

The total number of patient cases in the trauma emergency department was 417 during the shutdown period (35 days), 210 during the pre-shutdown period (15 days), 198 during post-shutdown period (15 days) and 575 patient cases during CTRL (35 days). This resulted in a lower number of daily cases during the shutdown period (11.94 ± 3.404) compared to CTRL (16.43 ± 4.907) with an IRR of 0.73 (shutdown period vs. CTRL, 95% CI [0.45; 1.18]). The comparison between daily cases during the pre-shutdown period (14.00 ± 4.629) and CTRL (16.43 ± 4.907) showed a decrease as well [IRR of 0.85; pre-shutdown vs. CTRL; 95% CI (0.46; 1.59)]. A further decrease of daily cases was found when comparing the pre-shutdown (14.00 ± 4.629) and the shutdown period (11.94 ± 3.404) with an IRR of 0.85 [pre-shutdown vs. shutdown; 95% CI (0.46; 1.6)]. Finally, daily cases increased again during the post-shutdown period [shutdown period 11.94 ± 3.404, post-shutdown period 13.13 ± 3.067 with an IRR of 1.1; post-shutdown vs. shutdown; 95% CI (0.59 ± 2.06)] (Table 1, Fig. 1).

**Table 1:** Total and daily numbers of total trauma patient cases sorted by defined time periods before, during and after the COVID-19 shutdown

	CTRL (35d)	PREST (15d)	SHUTDOWN (35d)	POST (15d)
Total cases (*n*)	575	210	417	198
Daily total cases (± SD)	16.43 (4.907)	14.00 (4.629)	11.94 (3.404)	13.13 (3.067)
IRR (95% CI)	SHUTDOWN vs. CTRL 0.73 (0.45; 1.18)	PREST vs. CTRL 0.85 (0.46; 1.59)	SHUTDOWN vs. PREST 0.85 (0.46; 1.6)	POST vs. SHUTDOWN 1.1 (0.59; 2.06)

IRRs are given for indicated time frame comparisons

*CTRL*  control period, *PREST*  pre-shutdown period, *SHUTDOWN*  shutdown, *POST*  post-shutdown period, *SD*  standard deviation, *IRR*  incidence rate ratio; 95% CI 95% confidence interval

To compare injury patterns, trauma mechanisms, cause of trauma and demographics, RR-values were calculated out of absolute numbers and incidence proportions during the CTRL and the shutdown period. Table 2 illustrates absolute numbers, incidence proportions and RR-values.

**Table 2:** Gender, trauma environments and cause, non-traumatic orthopedic presentation, trauma mechanism, treatment, admissions/discharges sorted by defined time periods before and during the COVID-19 shutdown

	CTRL (35d) with total n (incidence proportion: *n*/575 in %)	SHUTDOWN (35d) with total n (incidence proportion: *n*/417 in %)	SHUTDOWN vs. CTRL (95% CI)
All	575	417	
Gender			
Male	339 (59.0)	242 (58.0)	0.984 (0.885; 1.095)
Female	236 (41.0)	175 (42.0)	1.022 (0.881; 1.187)
Substance abuse			
Intoxication	3 (0.5)	4 (1.0)	1.839 (0.414; 8.171)
Alcohol	25 (4.3)	5 (1.2)	**0.276 (0.106; 0.714)**
Other	0	0	–
Serious injuries			
Death	5 (0.9)	9 (2.2)	2.482 (0.838; 7.352)
Polytrauma	9 (1.6)	7 (1.7)	1.072 (0.403; 2.857)
Traffic accidents			
Car accidents	21 (3.7)	12 (2.9)	0.788 (0.392; 1.583)
Bike accidents	51 (8.9)	30 (7.2)	0.811 (0.526; 1.251)
Scooter accidents	12 (2.1)	12 (2.9)	1.379 (0.626; 3.039)
Pedestrian accidents	15 (2.6)	9 (2.2)	0.827 (0.366; 1.872)
Public transport accidents	1 (0.2)	0	–
Workplace accidents			
Work injuries	69 (12.0)	59 (14.1)	1.179 (0.853; 1.630)
Work violence-related injuries	1 (0.2)	1 (0.2)	1.379 (0.086; 21.982)
Injuries in private environment			
Domestic violence-related injuries	0	1 (0.2)	
Violence-related injuries	22 (3.8)	9 (2.2)	0.564 (0.262; 1.212)
Robbery-related injuries	0	0	
Party-related injuries	13 (2.3)	0	
Sport injuries	114 (19.8)	41 (9.8)	**0.496 (0.355; 0.693)**
Home injuries	154 (26.8)	198 (47.5)	**1.773 (1.498; 2.099**)
Suicide attempts	0	4 (1.0)	
Selfharms	4 (0.7)	6 (1.4)	2.068 (0.587; 7.283)
Psychological disorders	2 (0.3)	2 (0.5)	1.379 (0.195; 9.749)
Non-traumatic orthopedic presentations			
General diseases	9 (1.6)	10 (2.4)	1.532 (0.628; 3.737)
Check up	42 (7.3)	13 (3.1)	**0.427 (0.232; 0.785)**
Trauma mechanisms			
Blunt	407 (70.9)	303 (72.7)	1.025 (0.947; 1.109)
Cut	39 (6.8)	57 (13.7)	**2.015 (1.368; 2.969)**
Penetration	24 (4.2)	28 (6.7)	1.609 (0.946; 2.734)
Shot	1 (0.2)	0	
Burn	4 (0.7)	11 (2.6)	**3.792 (1.216; 11.826)**
Bite	4 (0.7)	3 (0.7)	1.034 (0.233; 4.596)
Fall < 3 m	221 (38.4)	172 (41.2)	1.073 (0.920; 1.252)
Fall > 3 m	10 (1.7)	7 (1.7)	0.965 (0.370; 2.515)
Therapy			
Emergency surgery	47 (8.2)	51 (12.2)	**1.496 (1.028; 2.179)**
Semi-elective surgery	64 (11.1)	18 (4.3)	**0.388 (0.233; 0.644)**
Conservative treatment	368 (64.0)	344 (82.5)	**1.289 (1.195; 1.390)**
Admissions/discharges			
Ambulantory care	284 (49.4)	290 (69.5)	**1.408 (1.269; 1.563)**
Self discharged	17 (3.0)	18 (4.3)	1.460 (0.762; 2.799)
Stationary admission	262 (45.6)	100 (24.0)	**0.526 (0.434; 0.638)**
Discharged 24 h	33 (5.7)	27 (6.5)	1.128 (0.689; 1.847)
Discharged ≤ 5d	145 (25.2)	31 (7.4)	**0.295 (0.204; 0.425)**
Discharged ≤ 7d	23 (4.0)	10 (2.4)	0.600 (0.288; 1.246)
Discharged ≤ 1 month	55 (9.6)	30 (7.2)	0.752 (0.491; 1.152)
Discharged ≥ 1 month	7 (1.2)	2 (0.5)	0.394 (0.082; 1.887)
Send to other facility	17 (3.0)	20 (4.8)	1.622 (0.860; 3.059)

Total numbers and incidence proportions are given for defined time periods in first two columns and RRs show the comparison between the SHUTDOWN and CTRL, relative to trauma case numbers, in the third column. All RRs related to a CI not including 1 are highlighted in bold

*CTRL*  control, *SHUTDOWN*  shutdown, *RR*  risk ratio, 95% confidence interval

**Fig. 2:**
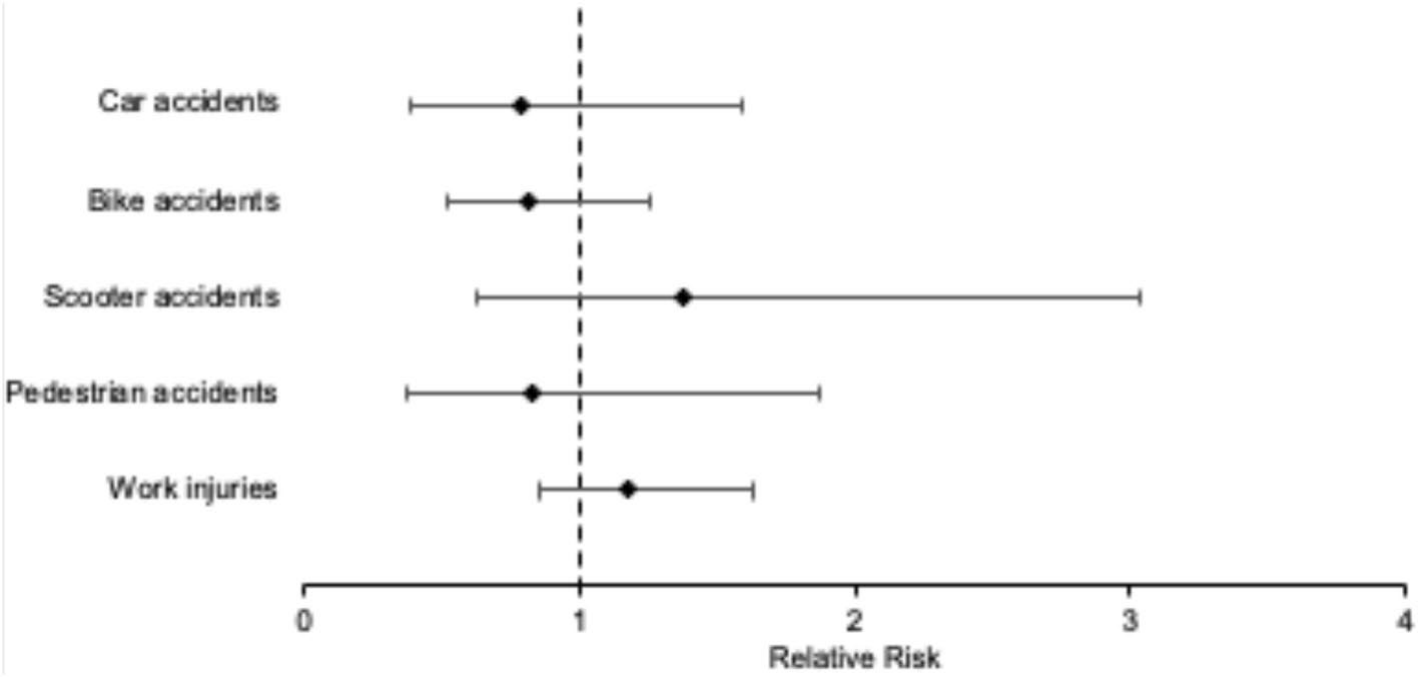
Forest plot of the relative risk for traffic and work accidents during the shutdown period: the relative risk of car, bike and pedestrian accidents decreased. The relative risk of scooter and work injuries were recorded with a slight increase during the shutdown period compared to CTRL

The absolute number of trauma-related death during CTRL was 5 and increased to 7 during the shutdown period. 9 polytrauma cases with an ISS of 32.5 were recorded during CTRL and 7 during the shutdown period with an average ISS of 34.1. Overall, traffic-associated accident decreased during the shutdown period. Figure 2 shows the relative risk in detail: the relative risk of car accidents decreased from 3.7% during CTRL to 2.9% during the shutdown, of bike accidents from 8.9 to 7.2% and of pedestrian accidents from 2.6 to 2.2% during mentioned time periods. The relative risk of scooter accidents (from 2.1 to 2.9%) and work injuries (from 12.0 to 14.1%) were recorded with a slight increase during the shutdown period compared to CTRL.

The number of work injuries decreased during the shutdown period. We also observed a reduction of the total patient number, which results in a calculated increasing relative risk for work injuries during the shutdown period.

Tables 2, 3 give detailed information concerning demographics and working background. Professional activities were documented with absolute numbers. Noticeable is a decrease in employment (98 during CTRL and 45 during shutdown), in office work, manual workman and students in contrast to an increase of injured medical staff (19 during CTRL and 29 during shutdown).

**Table 3:** Absolut numbers of type of employment sorted by defined time periods before and during the COVID-19 shutdown

	CTRL (35d)	SHUTDOWN (35d)
All	575	417
Office	7	1
Manual	43	15
Public	8	0
Medical	19	29
Student	20	0

**Fig. 3:**
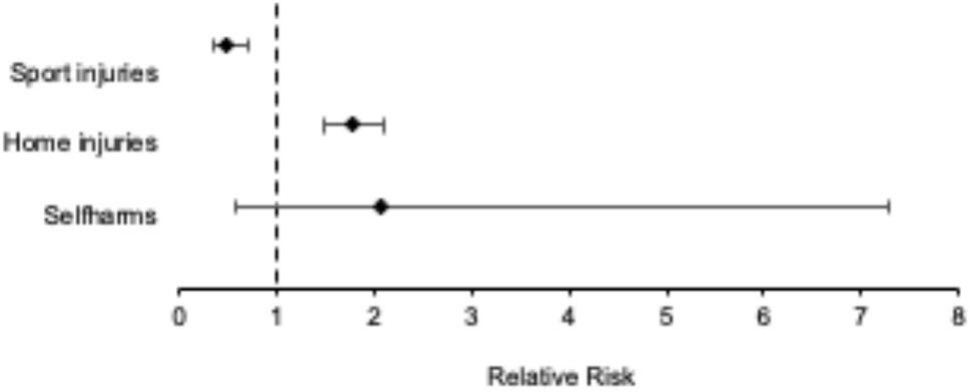
Forest plot of the relative risk for home, sport, psychological attempted injuries during the shutdown period with an increase concerning home and psychological attempted injuries and a decrease concerning sport injuries

The absolute number of household accidents increased from 154 during CTRL to 198 during the shutdown period with a corresponding increase of incidence proportion from 26.8 to 47.5%, which results in a RR-value of 1.773 [CTRL vs. shutdown period; 95% CI (1.498; 2.099)]. While the absolute number of sport injuries decreased from 114 (incidence proportion: 19.8%) during CTRL to 41 (incidence proportion: 9.8%) during the shutdown period with a decreasing incidence proportion and a resulting RR-value of 0.496 [CTRL vs. shutdown; 95% CI (0.355; 0.693)]. Four cases of suicide attempts were recorded (incidence proportion: 1.0%) during the shutdown period and no case during CTRL. Absolute numbers of self-harm increased slightly from 4 during CTRL to 6 during the shutdown period, resulting in incidence proportions of 0.7 an 1.4%. Comparable tendencies could be found concerning psychological disorders with increasing incidence proportions from 0.3 (CTRL) to 0.5% (shutdown) (Fig. 3).

**Fig. 4:**
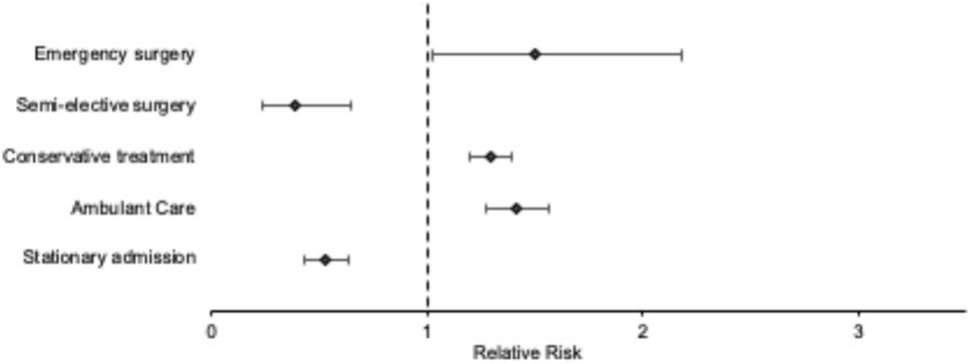
Forest plot of the relative risk of different treatment modalities during the shutdown period with an increase of emergency surgery and a decrease of elective surgery and hospitalization. During the shutdown period, the relative risk of conservative treatment and ambulant care increased

Figure 4 shows the relative risk for different treatment modalities. Absolute numbers of emergency surgery increased during the shutdown period with an increasing incidence proportion from 8.2 to 12.2% resulting in an RR of 1.496 [CTRL vs. shutdown; 95% CI (1.028; 2.179)]. Emergency surgery is defined as immediate surgery after admission and primary diagnosis. According to the requirements of the German government, elective surgery was reduced with incidence proportions of 11.1% during CTRL to 4.3% during shutdown period [RR of 0.388; CTRL vs. shutdown; 95% CI (0.233; 0.644)]. Accordingly, fewer patients were hospitalized (262 during CTRL vs. 100 during the shutdown) (Fig. 4).

Overall, absolute number of fractures decreased from 227 during CTRL to 161 during shutdown with a corresponding incidence proportion of 39.5–38.6%. Table 4 shows the distribution of fracture localizations. A noticeable and statistically significant increase of open fractures was recorded during shutdown period with an incidence proportion of 2.6% (0.9% during CTRL) and a RR-value of 3.034 [CTRL vs. shutdown; 95% CI (1.062; 8.665)]. Accordingly, the incidence proportion of open soft tissue injuries increased from 27.8 (CTRL) to 43.2% during shutdown period with a RR-value of 1.551 [CTRL vs. shutdown; 95% CI (1.307; 1.842)] (Table 4).

**Table 4:** Diagnosis of the trauma cases (fracture, soft tissue injury, brain injury) sorted by defined time periods before and during the COVID-19 shutdown

	CTRL (35d) with total n (incidence proportion: *n*/575 in %)	SHUTDOWN (35d) with total n (incidence proportion: *n*/417 in %)	SHUTDOWN vs. CTRL RR (95% CI)
All	575	417	
Fracture			
All fractures	227 (39.5)	161 (38.6)	
Facial fracture	29 (5.0)	19 (4.6)	0.903 (0.514; 1.589)
Skull fracture	11 (1.9)	3 (0.7)	0.376 (0.106; 1.340)
Clavicle fracture	14 (2.4)	11 (2.6)	1.083 (0.497; 2.362)
Humerus fracture	9 (1.6)	8 (1.9)	1.226 (0.477; 3.150)
Olecranon fracture	3 (0.5)	2 (0.5)	0.919 (0.154; 5.477)
Radius/ulnar fracture	21 (3.7)	21 (5.0)	1.379 (0.763; 2.491)
Hand fracture	32 (5.6)	20 (4.8)	0.862 (0.500; 1.485)
Thorax fracture	20 (3.5)	8 (1.9)	0.552 (0.245; 1.240)
Occipital/cervical spine fracture	13 (2.3)	6 (1.4)	0.636 (0.244; 1.661)
Cervical spine fracture	5 (0.9)	3 (0.7)	0.827 (0.199; 3.443)
Thoracic spine fracture	0	0	
Lumbar spine fracture	7 (1.2)	6 (1.4)	1.182 (0.400; 3.491)
Sacral spine fracture	3 (0.5)	4 (1.0)	1.839 (0.414; 8.171)
Pelvis fracture	9 (1.6)	12 (2.9)	1.839 (0.782; 4.323)
Femoral fracture	11 (1.9)	12 (2.9)	1.504 (0.670; 3.376)
Tibia/fibula fracture	14 (2.4)	14 (3.4)	1.379 (0.665; 2.861)
Patella fracture	6 (1.0)	1 (0.2)	0.230 (0.028; 1.902)
Foot fracture	20 (3.5)	11 (2.6)	0.758 (0.367; 1.566)
Open fracture	5 (0.9)	11 (2.6)	**3.034 (1.062; 8.665)**
Soft tissue injury			
Closed soft tissue injury	381 (66.4)	246 (59.0)	**0.889 (0.805; 0.981)**
Open soft tissue injury	160 (27.8)	180 (43.2)	**1.551 (1.307; 1.842)**
Bleeding soft tissue injury	131 (22.8)	130 (31.2)	**1.368 (1.112; 1.684)**
Brain injury			
SHT	68 (11.8)	43 (10.3)	0.872 (0.608; 1.250)
EDB	1 (0.2)	1 (0.2)	1.379 (0.086; 21.982)
SDB	8 (1.4)	4 (1.0)	0.689 (0.209; 2.274)
SAB	3 (0.5)	0	
ICB	2 (0.3)	2 (0.5)	1.379 (0.195; 9.749)
Trauma			
Thoracic trauma	4 (0.7)	6 (1.4)	2.068 (0.587; 7.283)
Pneumothorax	9 (1.6)	2 (0.5)	0.306 (0.067; 1.411)
Abdomentrauma	1 (0.2)	1 (0.2)	1.379 (0.086; 21.982)

Total numbers and incidence proportions are given for defined time periods in first two columns and RRs show the comparison between the SHUTDOWN and CTRL, relative to trauma case numbers, in the third column. All RRs related to a CI not including 1 are highlighted in bold

*CTRL*  control, *SHUTDOWN * shutdown, *RR*  risk ratio, 95% confidence interval

**Fig. 5:**
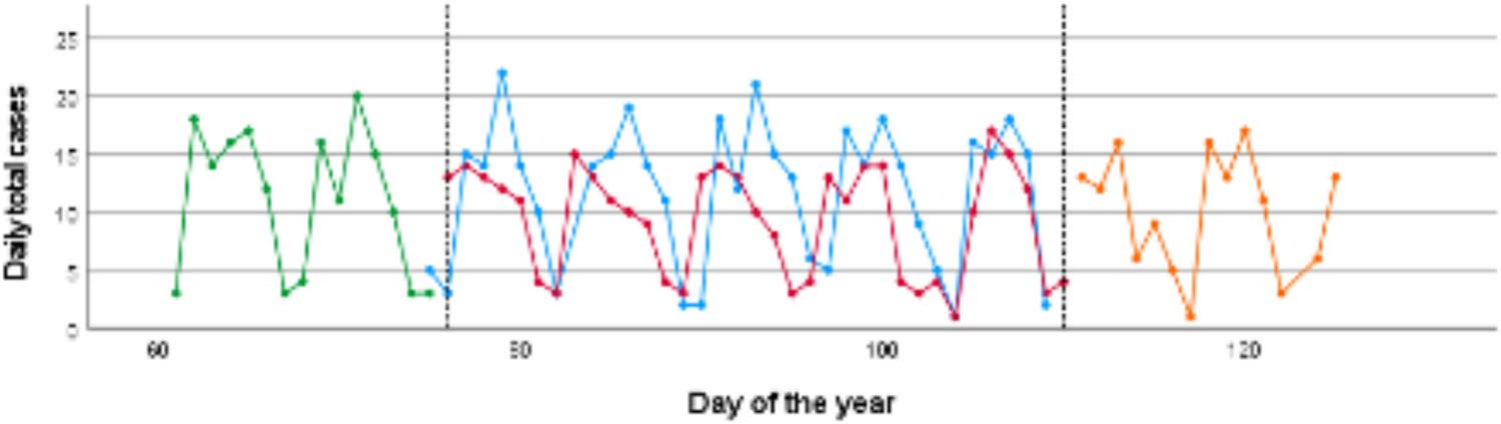
Development of daily total OP cases before, during and after the COVID-19 shutdown. The solid lines describe the development in the CTRL-period (blue) in 2019, pre-shutdown (green), shutdown (red) and post-shutdown (orange) period in 2020. Dotted lines indicate the beginning and end of the shutdown period (first line: Mar 16th 2020, second line: Apr 20th 2020)

397 surgeries were performed during CTRL compared to 325 during the shutdown period. The amount of daily operation decreased from 11.68 operations/day during CTRL to 9.29 operations/day during the shutdown period with an IRR of 0.8 [shutdown vs. CTRL; 95% CI (0.49; 1.30)] (Table 5, Fig. 5).

**Table 5:** Total and daily numbers of total OP patient cases sorted by defined time periods before, during and after the COVID-19 shutdown

	CTRL (35d)	PREST (15d)	SHUTDWON (35d)	POST (15d)
Totale cases (*n*)	397	165	325	141
Daily total cases (± SD)	11.68 (6.094)	11.00 (6.256)	9.29 (4.719)	10.07 (5.121)
IRR (95% CI)	SHUTDOWN vs. CTRL 0.80 (0.49;1.30)	PREST vs. CTRL 0.94 (0.5; 1.78)	SHUTDOWN vs. PREST 0.84 (0.45; 1.59)	POST vs. SHUTDOWN 1.08 (0.57; 2.08)

IRRs are given for indicated time frame comparisons

*CTRL*  control period, *PREST*  pre-shutdown period, *SHUTDOWN*  shutdown, *POST*  post-shutdown period, *SD*  standard deviation, *IRR*  incidence rate ratio, *95% CI * 95% confidence interval

**Fig. 6:**
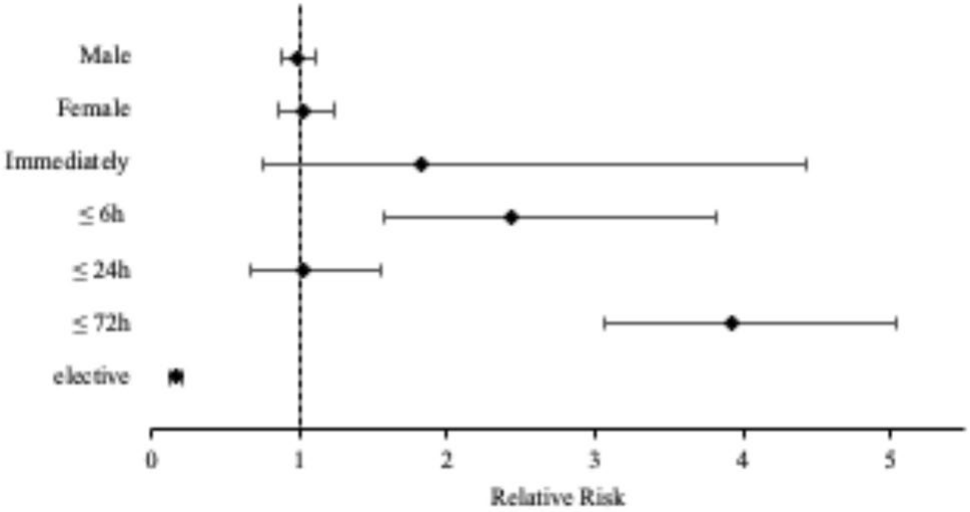
Forest plot of the relative risk of surgery during the shutdown period. During the shutdown period, an increase of high urgency surgeries and a decrease of elective surgery were recorded

According to requirements given by the German government, we found more high urgency surgeries compared to CTRL. A significant decrease of elective surgery was reported with 33 (10.2%) cases during the shutdown period compared to 261 (65.7%) cases during CTRL, which results in a RR-value of 0.154 [CTRL vs. shutdown; 95% CI (0.111; 0.215)]. The classification to an elective type of surgery was related to the indication. Due to certain reasons, such as decompensation of chronic disorders these surgeries were performed. Furthermore, an increase of all urgent operations according to the Amsterdam Criteria was found (Fig. 6, Table 6).

**Table 6:** Gender and priority of the surgeries sorted by defined time periods before and during the COVID-19 shutdown

	CTRL (35d) with total n (incidence proportion: *n*/397 in %)	SHUTDOWN (35d) with total n (incidence proportion: *n*/325 in %)	SHUTDOWN vs. CTRL RR (95% CI)
All	397	325	
Gender			
Male	244 (61.5)	197 (60.6)	0.986 (0.877; 1.109)
Female	153 (38.5)	128 (39.4)	1.022 (0.851; 1.228)
Serious injuries			
Death during hospital stay	6 (1.5)/0	6 (1.8)/2 (0.6)	
Polytrauma	3 (0.8)	9 (2.8)	
Surgery			
Immediate	8 (2.0)	12 (3.7)	1.832 (0.758; 4.429)
≤ 6 h	26 (6.5)	52 (16.0)	**2.443 (1.562; 3.821)**
≤ 24 h	42 (10.6)	35 (10.8)	1.018 (0.666; 1.555)
≤ 72 h	60 (15.1)	193 (59.4)	**3.929 (3.061; 5.045)**
Elective	261 (65.7)	33 (10.2)	**0.154 (0.111; 0.215)**

Total numbers and incidence proportions are given for defined time periods in first two columns and RRs show the comparison between the SHUTDOWN and CTRL, relative to OP case numbers, in the third column

All RRs related to a CI not including 1 are highlighted in bold

*CTRL*  control, *SHUTDOWN*  shutdown, *RR*  risk ratio, 95% confidence interval

In addition, we observed a reduction of total operation time of 18% during the shutdown period compared to CTRL. The Case Mix Index (CMI) as a measure for case severity, calculated by the hospital’s Diagnosis Related Group (DRG)-System, resulted in an increase of 0.32 points with a CMI = 2.32 during the shutdown period and CMI = 2.00 during CTRL.

## Discussion

Our study evaluates the impact of the COVID-19 shutdown in Germany on patient numbers, injury patterns and operation numbers on a German Level I trauma center. Overall, we observed a decrease of patient numbers in our emergency department: compared to a calendar-matched time period in 2019 patient, cases declined by about 30% (absolute numbers) during the shutdown period and by about 15% during the pre-shutdown period. In temporal connection, an increase of absolute numbers of patient cases of 10% was observed comparing the shutdown versus the post-shutdown period. A similar tendency was reported in an analysis by Christey et al. [12]. They documented a decrease of absolute admission numbers due to injury of 43% in a trauma center in New Zealand. In accordance, an American group analyzed average daily patient cases in a face and hand surgery department of a Level I trauma center in Chicago, Illinois 3 weeks before and 3 weeks after the “stay-at-home prescription”. They found a decrease of average daily cases from 4.2 to 2.9 [13].

We also evaluated the impact of the COVID-19 shutdown on operation numbers. Compared to 2019, we found the absolute numbers to be declined by about 5% during the pre-shutdown period and of about 20% during the shutdown period. The post-shutdown period showed an increase of absolute operation numbers of about 10%. A report of the Imperial College Healthcare NHS Trust and North West London Major Trauma Centre (Level I trauma center, London, UK) reveals a decrease of 1/3 of operation numbers in comparable time periods due to cancellation of semi-elective and elective surgery and underlines our findings [10]. A retrospective study from Italy goes in accordance with those data. This group showed a reduction of emergency surgery of 41.3% at the peak of pandemic shutdown (with comparable time periods) in a department of general surgery in the University Hospital of Ferrara, Italy, which is located in the Emilia Romagna—a region which was nearly most affected by the pandemic in Europe [14]. Both studies mentioned above describe a reduction of operation numbers in accordance to severity of local infection numbers and to country specific restrictions.

Concerning the operation numbers in detail, we can indicate a reduction of elective surgery of 85% during shutdown period compared to 2019 and a 2.5-fold increase of emergency surgery during the shutdown period, which is in contrast with the aforementioned findings from Italy. The significant increase of emergency surgeries might be explained through the local structure of regional trauma centers, where the shutdown and quarantine of medical staff affected the availability of these units.

Analysis of demographic data concerning professions shows a decrease of injuries in connection with office work, manual work and being a student. This can be explained by the governmental restrictions. In contrast, we found an increase of patient cases of medical staff. This might be explained by high medical workload and a need of necessary work protection. Haffner et al. already pointed out the need of appropriate supply of university hospitals in Germany [5]. Concerning the distribution of age, we observed a decrease of patient cases with the age 19–29 years, probably due to restrictions and quarantine.

With regard to trauma mechanisms, we found a clear reduction of alcohol intoxication/abuse of about 70% during shutdown. This was unexpected and contradict with findings from China. Evaluation of SARS-epidemic in 2003 shows and predicts an increase of alcohol consumption during epidemic events [15]. As expected, and in connection with quarantine and stay-at-home-rules, household accidents ascend up to 70% during the shutdown period in contrast to sport injuries, which decreased by about 50%. Our findings go in accordance with previous data from Park et al. This group found a decrease of comparable trauma mechanism of about 89% [10].

Our emergency department noted just one case of domestic violence during shutdown period with an uncertain number of unreported cases. This is unexpected as several studies alert to rising numbers of domestic violence in accordance to mental stress, social isolation, narrowness and due to fears [16, 17]. Furthermore, we documented four cases of suicide attempts during shutdown period. This was expected and goes in accordance with findings of Sher et al. This group underlines a rising tendency of suicide attempts during and after COVID-19 pandemic as a result of psychological (subsequent) damage. Quarantine, isolation and pandemic afflict general public and have a tendency to exacerbate exciting psychological diseases [18]. Here, prevention is necessary in cases of social isolation for whatever reason. Colleagues from Germany state the same tendencies and the recommendation of prevention [19]. In addition, our results show a slight decrease of traffic accidents, which can be explained by governmental restrictions and a high percentage of home office work.

Furthermore, we looked at injury patterns in detail. During the shutdown period, we observed a reduction of total number of fractures of about 30% in comparison to the defined control period in 2019. Our data go in accordance with reports of Turkey and the USA. Study groups could also show a reduction of fracture incidence of about 30% [20, 21]. Furthermore, these findings are in accordance with the results of two studies from Italy that also documented a decrease of the total amount of fractures during COVID-19 shutdown [22, 23]. We found no statistical relevant results concerning the location of the fractures. However, the number of open fractures was three times higher during shutdown period as it was during control time period in 2019.

In accordance to the restrictions of the German government, we recorded an increase of ambulant cases of 40% during shutdown period compared to 2019 and a decrease of hospitalization of 50% during shutdown period, which reflects the reduction of elective and semi-elective surgery.

Limitations of our study are the retrospective and monocentric character with a limit of case numbers and potential confounding factors. Further analysis of date and comparison to further Level I trauma centers are necessary. In addition, surrounding hospitals focused on COVID-19-patients and transferred others. A further limitation might be the choice of our control time period in 2019. The question remains, if this chosen time period is representative.

To conclude our findings, we saw a decrease of total patient numbers in an emergency department of a Level I trauma center and a decrease of total number of operations during shutdown period. Concurrently, we observed an increase of severe open fractures and emergency/urgent operations, which goes in accordance with the higher CMI-points and only a slight decrease of operation time (cut to suture). Furthermore, trauma mechanism changed with less traffic, sport and work accidents. Our findings seem to be related to restrictions of the German government and their aim to control the infection process and to assure medical care including intensive care capacities during shutdown. Our findings are important for resource planning during potential future shutdowns and will hopefully help for further planning concerning distribution of support and medical treatment.

## Data Availability

The manuscript, including related data, figures and tables has not been previously published and is not under consideration elsewhere.

## Code availability

Software applications were used properly.
